# High prevalence of undiagnosed comorbidities among adolescents with obesity

**DOI:** 10.1038/s41598-020-76921-6

**Published:** 2020-11-18

**Authors:** Karen S. W. Leong, Thilini N. Jayasinghe, Brooke C. Wilson, José G. B. Derraik, Benjamin B. Albert, Valentina Chiavaroli, Darren M. Svirskis, Kathryn L. Beck, Cathryn A. Conlon, Yannan Jiang, William Schierding, Tommi Vatanen, David J. Holland, Justin M. O’Sullivan, Wayne S. Cutfield

**Affiliations:** 1 Liggins Institute, University of Auckland, Auckland, New Zealand; 2 A Better Start-National Science Challenge, Auckland, New Zealand; 3 Department of Endocrinology, Children’s Hospital, Zhejiang University School of Medicine, Hangzhou, China; 4 Department of Women’s and Children’s Health, Uppsala University, Uppsala, Sweden; 5 Neonatal Intensive Care Unit, Pescara Public Hospital, Pescara, Italy; 6 School of Pharmacy, Faculty of Medical and Health Sciences, University of Auckland, Auckland, New Zealand; 7 School of Sport, Exercise and Nutrition, College of Health, Massey University, Auckland, New Zealand; 8 Department of Statistics, University of Auckland, Auckland, New Zealand; 9 Broad Institute of MIT and Harvard, Cambridge, MA USA; 10 Department of Infectious Diseases, Counties Manukau District Health Board, Auckland, New Zealand

**Keywords:** Diseases, Endocrinology

## Abstract

Metabolic diseases are increasing among adolescents with obesity. Although the reported prevalence of metabolic syndrome is approximately 30% worldwide, its prevalence is largely unknown among New Zealand adolescents. Therefore, we assessed the health of adolescents with obesity (BMI ≥ 30 kg/m^2^) enrolled in a randomised clinical trial (Gut Bugs Trial), to identify the prevalence of undiagnosed comorbidities. Assessments included anthropometry, 24-h ambulatory blood pressure monitoring, and insulin sensitivity. We report on baseline data (pre-randomisation) on 87 participants (14–18 years; 59% females), with mean BMI 36.9 ± 5.3 kg/m^2^ (BMI SDS 3.33 ± 0.79). Approximately 40% of participants had undiagnosed metabolic syndrome, which was twice as common among males. Half (53%) had pre-diabetes and 92% a reduction in insulin sensitivity. Moreover, 31% had pre-hypertension/hypertension, 69% dyslipidaemia, and 25% abnormal liver function. Participants with class III obesity had a greater risk of metabolic syndrome than those with classes I/II [relative risk 1.99 (95% CI 1.19, 3.34)]. Risks for pre-hypertension/hypertension and inflammation were also greater among those with class III obesity. We identified a high prevalence of undiagnosed comorbidities among adolescents with obesity in New Zealand. As adolescent obesity tracks into adulthood, early interventions are needed to prevent progression to overt cardiometabolic diseases.

## Introduction

With over 120 million affected children and adolescents worldwide, paediatric obesity has become one of the largest health concerns of the modern world^1^. In 2016, the World Health Organization (WHO) estimated that, globally, the prevalence of paediatric overweight and obesity was 18%^2^. In New Zealand, an even higher rate was reported for the same year, whereby nearly 40% had overweight or obesity (> 16% with obesity)^3^. In 2019, 22% of New Zealand adolescents were overweight while 12% had obesity^4^, which is higher than in many parts of the world such as Australia (combined rate of overweight/obesity is 25%)^5^, Denmark (22%)^6^, and China (26%)^7^ but lower than the US (40%)^8^. While the prevalence of paediatric obesity seems to have plateaued in many countries^9^ as in New Zealand^10^, the prevalence of adolescents with obesity remains high in New Zealand, particularly among Māori (New Zealand's indigenous people) and Pacific adolescents, and those from areas of greater socioeconomic deprivation^4,11^. Of note, within a 5-year period from 2007 to 2012, there was a rapid increase in the prevalence of Pacific adolescents with obesity (from 27% to 34%) and severe obesity (9% to 14%)^11^.

Adolescence is a period of accelerated growth characterised by rapid physiological, hormonal, and developmental changes, with marked alterations in body composition and weight gain^12^. There are changes in the hormonal regulation of appetite and satiety in both sexes, as well as increases in adiposity and changes in fat distribution among females, which contribute to a tendency to gain weight in adolescence^12,13^. For some adolescents, the normal changes observed in puberty can be magnified, leading to greater weight gain and metabolic dysfunction, including persistence of insulin resistance^14^. As 90% of adolescents with obesity continue to have obesity as adults^15^, early intervention is crucial.

Consistent with the rise in obesity, cardiometabolic comorbidities such as metabolic syndrome and type 2 diabetes mellitus (T2DM) are increasing in children and adolescents^16^. Increasing body mass index (BMI) is associated with an increased risk of metabolic syndrome^17^, which includes increased abdominal obesity, hypertension, impaired fasting glycaemia, dyslipidaemia, and is associated with insulin resistance^16^. The prevalence of metabolic syndrome among adolescents with obesity has been reported to be as high as 60%^18^, and it is associated with the development of T2DM^19^, cardiovascular diseases^20^, and a two-fold increase in the risk of coronary artery disease and stroke, and a 1.5-fold increase in the risk of all-cause mortality^21^. In the US, a national cross-sectional study reported that T2DM is increasingly diagnosed among adolescents and accounted for 40% of adolescent diabetes, with more than a third of T2DM cases undiagnosed prior to the study^22^. In New Zealand, the number of children with T2DM is increasing at approximately 5% per year, and this disease disproportionally affects high-risk ethnic groups (Māori and Pacific)^23^. Apart from serious cardiometabolic complications, paediatric obesity has been associated with increased mortality even in early adulthood^24^. This is likely due to increased systemic inflammation, insulin resistance, impaired cardiovascular function, and the development of non-alcoholic fatty liver disease^25,26^. Moreover, many of these children face bullying^27^ and social isolation^28^, as well as increased rates of depression^29^, suicide and self-harm^24^.

Overall, there are limited data on the prevalence of obesity-related comorbidities among adolescents with obesity in New Zealand^30,31^. Due to the numerous complications associated with obesity, early identification particularly in high-risk populations is necessary so that targeted interventions can be implemented. Therefore, we aimed to assess the metabolic health of a group of adolescents with obesity enrolled in a clinical trial and identify the prevalence of undiagnosed metabolic syndrome and other obesity-related cardiometabolic comorbidities.

## Methods

### Ethics

This study reported on baseline data (pre-randomisation) from a randomised placebo-controlled trial (Gut Bugs Trial) to evaluate the effectiveness of faecal microbiome transfer for treatment of adolescent obesity in Auckland, New Zealand^32^. The trial was registered with the Australian New Zealand Clinical Trials Registry (ACTRN12615001351505); ethics approval was granted by the Northern A Health and Disability Ethics Committee (16/NTA/172). Participants provided verbal and written informed consents. All procedures in this study were conducted according to the ethical principles and guidelines laid down in the Declaration of Helsinki^33^.

### Recruitment

Participants were recruited from social media through Facebook advertisements between 2017–2018. All were post-pubertal, aged 14–18 years, with BMI ≥ 30 kg/m^2^, who were not diagnosed with diabetes or chronic diseases that could affect weight or metabolism^32^.

### Clinical assessments

Clinical assessments included medical and physical examinations previously described in the trial’s protocol^32^, and briefly summarised here.

Height, weight, and waist and hip circumferences were measured^32^. BMI values were converted into standard deviation score (SDS) using WHO standards^34^. For comparison within our study population, BMI was stratified using the adult criteria for obesity: Class I (BMI ≥ 30 but < 35 kg/m^2^); Class II (≥ 35 but < 40 kg/m^2^); and Class III (≥ 40 kg/m^2^)^35^. Body composition was assessed using whole-body dual-energy X-ray absorptiometry (DXA; Lunar Prodigy and Lunar iDXA; GE Medical Systems, Chicago, Illinois, USA).

Clinic resting systolic and diastolic blood pressures (BP) were measured using an automated BP monitor (Ri-champion N; Riester, Jungingen, Germany). 24-h ambulatory BP monitoring (24hABPM) was performed using an oscillometric device (Spacelabs OnTrak; Spacelabs Medical Inc, Redmond, Washington, USA).

Participants underwent a 75-g oral glucose tolerance test (OGTT)^32^. Insulin sensitivity was assessed by homeostatic model assessment of insulin resistance (HOMA-IR)^36^ and Matsuda index^37^, as previously described^32^. Other key markers of glucose metabolism measured were fasting insulin and fasting glucose, 2-h glucose, and glycated haemoglobin (HbA1c). From fasting blood samples, uric acid, high-sensitivity C-reactive protein (hsCRP), lipid profile, and liver function were measured^32^.

Health outcomes in this study were cardiometabolic comorbidities as defined in Table 1.

**Table 1:** Definitions of cardiometabolic comorbidities.

Assessments		Comorbidities	Thresholds for abnormal results	References
Waist circumference		Elevated waist circumference	14 years: ≥ 90th percentile (≥ 79.9 cm for males; ≥ 77 cm for females)	Zimmet et al. 2007^16^; Eisenmann et al. 2005^63^
			15 years: ≥ 90th percentile (≥ 81.7 cm for males; ≥ 78.4 cm for females)	
			≥ 16 years: ≥ 94 cm for males and ≥ 80 cm for females	
Glucose homeostasis		Elevated fasting glucose	Fasting blood glucose ≥ 5.6 mmol/L	American Diabetes Association 2018^53^; Frithioff‐Bøjsøe et al. 2019^64^
		Elevated 2-h glucose (OGTT)	2-h blood glucose ≥ 7.8 mmol/L	
		Elevated HbA1c	HbA1c ≥ 39 mmol/mol	
			HbA1c ≥ 5.7%	
		Elevated fasting insulin	< 15 years: > 11.4 µU/mL for males and > 14.0 µU/mL for females	
			≥ 15 years: > 11.4 µU/mL for males and > 12.9 µU/mL for females	
		Pre-diabetes	Fasting glucose ≥ 5.6 but < 7.0 mmol/L; 2-h glucose ≥ 7.8 but < 11.1 mmol/L; HbA1c ≥ 39 but < 48 mmol/mol	
		Diabetes	Fasting glucose ≥ 7.0 mmol/L; 2-h glucose ≥ 11.1 mmol/mol; HbA1c ≥ 48 mmol/mol	
Insulin resistance^a^		High HOMA-IR	HOMA_IR > 3.16	Keskin et al. 2005^39^
		Low Matsuda index	Matsuda index ≤ 2.5	Kernan et al. 2003^40^
Blood pressure	Clinic BP	Pre-hypertension	< 16 years: SBP and/or DBP ≥ 90th but < 95th percentile for age and sex	Lurbe et al. 2016^62^
			≥ 16 years: SBP ≥ 130 but < 140 mmHg and/or DBP ≥ 85 but < 90 mmHg	
		Hypertension	< 16 years: SBP and/or DBP ≥ 95th percentile for age and sex	
			≥ 16 years: SBP and/or DBP ≥ 140/90 mmHg	
	24hABPM	Pre-hypertension	SBP and/or DBP ≥ 90th but < 95th percentile for age and sex	
		Hypertension	SPB and/or DBP ≥ 95th percentile for sex, age, and height, unless BP is equal to or higher than adult criteria thresholds (i.e. mean 24 hr 130/80 mmHg; awake 135/85 mmHg; and sleep 125/75 mmHg)	
		Non-dippers	Nocturnal drop in SBP and/or DBP ≤ 10%	
Lipid profile		Low HDL	< 16 years: < 1.03 mmol/L	Zimmet et al. 2007^16^
			≥ 16 years: males < 1.03 mmol/L; females < 1.29 mmol/L	
		High LDL	> 2.6 mmol/L	NCEP 2001^65^
		High triglycerides	≥ 1.7 mmol/L	Zimmet et al. 2007^16^
		High total cholesterol	> 5.2 mmol/L	European Atherosclerosis Society 1987^66^
		Dyslipidaemia	Low HDL or high LDL or high triglycerides or high total cholesterol	
Inflammatory markers	Uric acid	Hyperuricaemia	Males ≥ 417 µmol/L; females ≥ 340 µmol/L	Thefeld et al. 1973^67^
	hsCRP	Elevated hsCRP	< 16 years: > 2.8 mg/L	Schlebusch et al. 2002^68^
			≥ 16 years ≥ 5.0 mg/L	Dati et al. 1996^69^
Liver function		Elevated ALT	Males > 41 U/L; females > 33 U/L	Klein et al. 1994^70^
		Elevated AST	Males > 40 U/L, females > 32 U/L	Thefeld et al. 1974^71^
		Elevated GGT	Males ≥ 60 U/L, females ≥ 40 U/L	Thomas et al. 2005^72^
		Abnormal liver function	Elevated ALT or elevated AST or elevated GGT	
Metabolic health		Metabolic syndrome	≥ 10 but < 16 years: Waist circumference ≥ 90th percentile (or adult cut-off if the latter is lower); AND any 2 of the following 4 criteria: 1. triglycerides ≥ 1.7 mmol/L 2. HDL < 1.03 mmol/L 3. SBP ≥ 130 and/or DBP ≥ 85 mmHg 4. Fasting glucose ≥ 5.6 mmol/L and/or previously diagnosed type 2 diabetes	Zimmet et al. 2007^16^
			≥ 16 years: Waist circumference ≥ 94 cm for males and ≥ 80 cm for females; AND any 2 of the following 4 criteria: 1. triglycerides ≥ 1.7 mmol/L 2. HDL < 1.03 mmol/L in males and < 1.29 mmol/L in females; or specific treatment for these lipid abnormalities 3. SBP ≥ 130 mmHg and/or DBP ≥ 85 mmHg, or treatment for previously diagnosed hypertension 4. Fasting glucose ≥ 5.6 mmol/L and/or previously diagnosed type 2 diabetes	

^a^The HOMA-IR cut-off of 3.16 was established from a group of adolescents^39^ and the Matsuda index cut-off of 2.5 was established from a group of healthy adults^40^.

*24hABPM* 24-h ambulatory blood pressure monitoring, *ALT* alanine transaminase, *AST* aspartate transaminase, *BP* blood pressure, *DBP* diastolic blood pressure, *GGT* gamma-glutamyl transferase, *hsCRP* high-sensitivity C-reactive protein, *HbA1c* haemoglobin A1c, *HDL* high-density lipoprotein cholesterol, *HOMA-IR* homeostatic model assessment of insulin resistance, *LDL* low-density lipoprotein cholesterol, *SBP* systolic blood pressure.

### Assays

Insulin levels were measured by electrochemiluminescence immunoassay (ECLIA) on the Roche Cobas e411 analyser (Roche, Basel, Switzerland) with a coefficient of variation (CV) of 1.2%. Glucose, HbA1c, uric acid, hsCRP, lipid profile, and liver function were measured on the Roche/Hitachi Cobas e311 (Roche) with CVs 4.1–6.8%.

### Data analyses

Data were analysed using SPSS v25 (IBM Corp, Armonk, NY, USA) and SAS v9.4 (SAS Institute, Cary, NC, USA). Baseline data were summarised as mean ± standard deviation (SD), median [quartile 1, quartile 3], or n (%), as appropriate. Differences in prevalence between obesity classes and sexes were examined with Chi-square tests or Fisher's exact tests, as appropriate. The likelihood of given comorbidities in participants with class III obesity was assessed with generalized linear regression models, using PROC GENMOD (SAS), adjusting for sex, and relative risk estimation by Poisson regression with robust error variance, and a log link^38^. The results were reported as relative risks (RR) with respective 95% confidence intervals (95% CI). Statistical tests were two-tailed, with significance levels maintained at p < 0.05.

## Results

### Participants

565 participants responded to advertisements; 328 (58%) were not eligible and 150 (27%) declined to participate. Thus, 87 participants (59% females) were recruited at a median age of 17.6 years (Table 2). 44% of our cohort were Māori or Pacific, and nearly 30% were from the most-deprived quintile of socioeconomic deprivation (Table 2). Their mean BMI was 36.9 kg/m^2^ (range 31.6–42.3 kg/m^2^), with mean BMI SDS 3.33 (range 2.10–6.38); 33%, 38%, and 29% of participants were classified as obesity class I, II, and III, respectively (Table 2). Mean total body fat was approximately 50% (Table 2).

**Table 2:** Demographic and clinical characteristics of participants enrolled into the Gut Bugs Trial.

	All	Females	Males
N	87 (100%)	51 (59%)	36 (41%)
Age (years)	17.6 [16.2, 18.3]	17.7 [16.2, 18.3]	16.9 [15.9, 18.2]
**Ethnicity**			
New Zealand European	43 (49%)	22 (43%)	21 (58%)
Māori	18 (21%)	12 (24%)	6 (17%)
Pacific	20 (23%)	13 (26%)	7 (19%)
Asian	6 (7%)	4 (8%)	2 (6%)
**Any current drug use**			
Tobacco smoking	8 (9%)	3 (6%)	5 (14%)
Alcohol	34 (39%)	25 (49%)	9 (25%)
**Socioeconomic deprivation**^b^			
Quintile 1 (least deprived)	6 (7%)	3 (6%)	3 (8%)
Quintile 2	20 (23%)	8 (16%)	12 (33%)
Quintile 3	22 (25%)	13 (25%)	9 (25%)
Quintile 4	15 (17%)	13 (25%)	2 (6%)
Quintile 5 (most deprived)	24 (28%)	14 (28%)	10 (28%)
**Anthropometry**			
Height (cm)	172.6 ± 8.6	168.1 ± 6.3	178.9 ± 7.3
Weight (kg)	112.6 ± 20.1	105.4 ± 15.7	122.9 ± 21.3
Waist circumference (cm)	106 ± 12	101 ± 8	113 ± 12
Waist-to-height ratio	0.61 ± 0.06	0.60 ± 0.04	0.63 ± 0.07
Waist-to-hip ratio	0.87 ± 0.08	0.82 ± 0.04	0.93 ± 0.08
BMI (kg/m^2^)	36.9 ± 5.3	36.1 ± 4.4	37.9 ± 6.4
BMI SDS	3.33 ± 0.79	3.17 ± 0.63	3.55 ± 0.94
Class I obesity	29 (33%)	15 (29%)	14 (39%)
Class II obesity	33 (38%)	23 (45%)	10 (28%)
Class III obesity	25 (29%)	13 (26%)	12 (33%)
**Body composition**			
Total body fat (%)	47.5 ± 5.6	50.0 ± 4.8	44.0 ± 4.9
**Insulin sensitivity**			
HOMA-IR	7.88 ± 5.53	7.21 ± 5.66	8.84 ± 5.27
Matsuda index	1.73 ± 1.13	1.99 ± 1.34	1.38 ± 0.63
**Maternal characteristics**			
Education (higher)^a^	55 (70%)	31 (67%)	24 (75%)
BMI (kg/m^2^)	33.7 ± 7.8	33.2 ± 8.0	34.4 ± 7.5
Class 1 obesity	26 (33%)	14 (30%)	12 (36%)
Class 2 obesity	11 (14%)	8 (17%)	3 (9%)
Class 3 obesity	16 (20%)	7 (15%)	9 (27%)
**Paternal characteristics**			
Education (higher)^a^	50 (63%)	26 (55%)	24 (75%)
BMI (kg/m^2^)	32.0 ± 5.5	31.4 ± 5.2	32.7 ± 5.7
Class 1 obesity	19 (27%)	13 (34%)	6 (18%)
Class 2 obesity	15 (21%)	5 (13%)	10 (30%)
Class 3 obesity	6 (9%)	3 (8%)	3 (9%)

Age data are median [quartile 1, quartile 3]; other data are n (%) or means ± SD, as appropriate.

*BMI* body mass index, *HOMA-IR* homeostatic model assessment of insulin resistance, *SDS* standard deviation score.

Obesity classes were defined as: Class I (BMI ≥ 30 kg/m^2^ but < 35 kg/m^2^); Class II (BMI ≥ 35 kg/m^2^ but < 40 kg/m^2^); and Class III (BMI ≥ 40 kg/m^2^).

^a^Higher maternal/paternal education status refer to university degree or post-high-school vocational qualification.

^b^Socioeconomic deprivation was estimated using the New Zealand Indices of Multiple Deprivation^73^.

### Comorbidities

There was a high prevalence of undiagnosed comorbidities (Table 3). Notably, one in three participants (36%) had undiagnosed metabolic syndrome (Table 3), with this condition twice as common among males (50% vs 26%; p = 0.018).

**Table 3:** Baseline cardiometabolic comorbidities of adolescents with obesity enrolled into the Gut Bugs Trial.

Assessments	Cardiometabolic comorbidities^a^	All	Females	Males
N		87	51	36
Anthropometry	Elevated waist circumference	87 (100%)	51 (100%)	36 (100%)
Clinic blood pressure	Pre-hypertension	11 (13%)	4 (8%)	7 (19%)
	Hypertension	7 (8%)	4 (8%)	3 (8%)
24hABPM	Awake pre-hypertension	2 (2%)	1 (2%)	1 (3%)
	Awake hypertension	4 (5%)	3 (6%)	1 (3%)
	Asleep pre-hypertension	16 (18%)	12 (24%)	4 (11%)
	Asleep hypertension	9 (10%)	5 (10%)	4 (11%)
	Any time pre-hypertension	15 (17%)	11 (22%)	4 (11%)
	Any time hypertension	12 (14%)	7 (14%)	5 (14%)
	Non-dippers (systolic)	43 (49%)	25 (49%)	18 (50%)
	Non-dippers (diastolic) ^b^	18 (21%)	11 (22%)	7 (19%)
Glucose metabolism	Elevated fasting glucose	29 (34%)	12 (24%)	17 (47%)
	Elevated 2-h glucose (OGTT)	10 (12%)	5 (10%)	5 (14%)
	Elevated HbA1c	27 (31%)	1 (2%)	26 (74%)
	Elevated fasting insulin	80 (94%)	44 (90%)	36 (100%)
	Pre-diabetes	44 (52%)	14 (29%)	30 (83%)
	Diabetes	1 (1%)	1 (2%)	0 (0%)
Insulin resistance	High HOMA-IR	78 (92%)	42 (86%)	36 (100%)
	Low Matsuda index	72 (87%)	37 (79%)	35 (97%)
Lipid profile	High total cholesterol	16 (19%)	10 (20%)	6 (17%)
	High LDL	46 (54%)	28 (56%)	18 (50%)
	Low HDL	37 (43%)	20 (40%)	17 (47%)
	High triglycerides	17 (20%)	4 (8%)	13 (36%)
	Dyslipidaemia	64 (74%)	37 (74%)	27 (75%)
Liver function	Elevated ALT	11 (13%)	6 (12%)	5 (14%)
	Elevated AST	15 (17%)	10 (20%)	5 (14%)
	Elevated GGT	11 (13%)	6 (12%)	5 (14%)
	Abnormal liver function	22 (25%)	14 (28%)	8 (22%)
Inflammatory markers	Hyperuricaemia	53 (61%)	34 (67%)	19 (53%)
	Elevated hsCRP	24 (28%)	15 (29%)	9 (25%)
Metabolic health	Metabolic syndrome	31 (36%)	13 (26%)	18 (50%)

Data are n (%).

^a^For the full definitions of all comorbidities please refer Table 1.

^b^All diastolic non-dippers were also systolic non-dippers.

*24hABPM* 24-h ambulatory blood pressure monitoring, *ALT* alanine transaminase, *AST* aspartate transaminase, *BP* blood pressure, *GGT* gamma-glutamyl transferase, *HbA1c* haemoglobin A1c, *HDL* high-density lipoprotein cholesterol, *HOMA-IR* homeostatic model assessment of insulin resistance, *hsCRP* high-sensitivity C-reactive protein, *LDL* low-density lipoprotein cholesterol, *OGTT* oral glucose-tolerance test.

In addition, 13% of participants had pre-hypertension and 8% had hypertension from clinic BP. From 24hABPM data, 17% were pre-hypertensive and 14% hypertensive with nocturnal pre-hypertension recorded in 18% and nocturnal hypertension in 10% of participants (Table 3).

Pre-diabetes was common, affecting approximately half of participants (52%): 29% females and 83% males (p < 0.0001). Fasting insulin was elevated in 94% of participants including all the males (Table 3). Most participants displayed a reduction in insulin sensitivity, as 92% had a high HOMA-IR when compared to a cohort of adolescents^39^, and 87% had a low Matsuda index when compared to healthy adults^40^ (Table 4).

**Table 4:** Relative risks of comorbidities among participants according to their obesity class.

Comorbidities^a^	Classes I/II	Class III	p1	Relative risk	p2
N	62	25			
Pre-hypertension or hypertension	11 (18%)	16 (64%)	** < 0.0001**	3.76 (2.06, 6.85)	** < 0.0001**
Awake pre-hypertension or hypertension	1 (2%)	5 (20%)	**0.0069**	13.02 (1.66, 102.01)	**0.015**
Sleep pre-hypertension or hypertension	11 (18%)	14 (56%)	** < 0.001**	3.30 (1.76, 6.18)	** < 0.001**
Systolic and diastolic non-dippers	8 (13%)	10 (40%)	**0.0048**	3.16 (1.41, 7.09)	**0.0053**
Pre-diabetes or diabetes	30 (48%)	15 (65%)	0.17	1.19 (0.83, 1.70)	0.35
Dyslipidaemia	45 (73%)	19 (79%)	0.53	1.09 (0.84, 1.41)	0.51
Abnormal liver function	17 (27%)	5 (20%)	0.47	0.74 (0.31, 1.79)	0.51
Hyperuricaemia	34 (55%)	19 (76%)	0.067	1.42 (1.03, 1.95)	**0.031**
Elevated hsCRP	13 (21%)	11 (44%)	**0.030**	2.14 (1.12, 4.11)	**0.022**
Metabolic syndrome	17 (27%)	14 (58%)	**0.0074**	1.99 (1.19, 3.34)	**0.0091**

Data are n (%), or relative risks (adjusted for sex) and respective 95% confidence intervals.

P-values for statistically significant differences are shown in bold.

Obesity classes were defined as: Class I (BMI ≥ 30 but < 35 kg/m^2^); Class II (≥ 35 but < 40 kg/m^2^); and Class III (≥ 40 kg/m^2^).

All blood pressure parameters were derived from 24-h ambulatory blood pressure monitoring.

*hsCRP* high-sensitivity C-reactive protein.

^a^For the full definitions of comorbidities please refer to Table 1.

Dyslipidaemia and abnormal liver function affected 74% and 25% of participants respectively (Table 3). Inflammatory markers were elevated, with 61% having hyperuricaemia and 28% with elevated hsCRP (Table 3).

### BMI classes

There were marked differences in the prevalence of cardiometabolic comorbidities between obesity classes (Table 4). The risk of metabolic syndrome increased among those with class III obesity compared to those with a lesser degree of obesity [RR 1.99 (95% CI 1.19, 3.34); p = 0.0091] (Table 4). The prevalence of BP abnormalities was markedly higher in participants with class III obesity, with the relative risks of pre-hypertension/hypertension and loss of the nocturnal dipping BP status more than 3 times greater in this group (Table 4). A higher BMI was associated with an increased likelihood of inflammation, with the relative risk of hyperuricaemia and elevated hsCRP being 1.4 and 2.1 times greater in participants with class III obesity, respectively (Table 4).

## Discussion

We identified a high prevalence of undiagnosed comorbidities amongst our cohort of adolescents with obesity. Notably, more than a third were diagnosed with metabolic syndrome, which was twice as common in males than in females. More than half (52%) of our cohort had pre-diabetes and more than 90% had fasting hyperinsulinaemia, with higher rates of these complications in males. In addition, almost all had a reduction in insulin sensitivity. Moreover, increased levels of adiposity were associated with a higher risk of metabolic syndrome, hypertension, and inflammation. The presence of these adverse cardiometabolic outcomes at a relatively young age is alarming, and along with published data documenting the tracking of weight-related comorbidities from childhood into adulthood^41^, further reaffirms that obesity in adolescence is far from a benign condition.

A description of comorbidities among 239 children and adolescents with obesity in New Zealand was provided by Anderson et al. in 2016^30^. In that study, 1 in 10 had elevated blood pressure, 1 in 4 had increased inflammation, and nearly half had dyslipidaemia and abnormal liver function^30^. While their reported prevalence of obesity-related comorbidities were relatively high, they were lower than those observed in the present study^30^, probably because their study population was younger (mean age 10.7 vs 17.2 years in our study), leaner (mean BMI 3.09 vs 3.33 SDS), and had a different ethnic make-up with a much lower representation from those of Pacific descent (3% vs 23%) than ours.

Worldwide, the reported prevalence of metabolic syndrome among children and adolescents with obesity varied between 10 to 66%^18,31,42–50^. In New Zealand, Grant et al. reported a lower rate of metabolic syndrome among 29 Pacific adolescents with obesity aged 15–18 years^31^ – 21% vs 36% in our study. In comparison, reported rates of metabolic syndrome in adolescents with obesity vary widely across the world: 15% to 50% in the US^18,50^, 23% to 60% in Latin America^18,43,46,49^, 12% to 42% in Asia^18^, and 14% to 44% in Europe^18,44,45,47^. The marked differences in prevalence among these studies could be attributed to variations in age distribution and ethnic composition of the respective study populations, as well as the definitions of metabolic syndrome used. Nonetheless, the findings from two systematic reviews clearly show increasing BMI as an important risk factor associated with the development of metabolic syndrome^18,42^, with this relationship also shown to occur at the upper end of the BMI spectrum by our stratified analyses.

Reduction in insulin sensitivity as well as impaired glucose metabolism were common complications among our study population. Insulin resistance as assessed from the HOMA-IR values among our adolescents was more than 1.5 times higher when compared with adolescents with obesity in the US^51^ and Europe^52^. In addition, more than half of our participants had pre-diabetes (i.e. impaired fasting glycaemia, impaired glucose tolerance, and/or elevated glycated haemoglobin). It could be argued that our high rate of pre-diabetes could be attributed, at least in part, to our lower cut-off value for impaired fasting glycaemia (i.e. ≥ 5.6 mmol/L as recommended by the ADA^53^ and ISPAD^54^, rather than the WHO value ≥ 6.1 mmol/L^55^), as using the higher WHO cut-off, our pre-diabetes rate would have dropped from 52 to 38%. Nonetheless, when compared to previous studies in US and Europe that used the same cut-off values as ours, the prevalence of pre-diabetes in our study was still 4 times greater^52,56^. Moreover, due to the high risk of diabetes in our vulnerable study population and our aim to prevent worsening of their metabolic health through early identification and intervention, we contend that the lower threshold for abnormal fasting glycaemia was justified. Nichols et al. reported that without appropriate intervention, nearly one in ten adults with pre-diabetes will develop T2DM within 3.5 years, and the progression to T2DM could be accelerated by risk factors such as increased BMI, elevated blood pressure and triglyceride levels, and lower HDL levels, all of which were present in our participants^57^. As improvement in insulin sensitivity and reversal of pre-diabetes have been reported with therapeutic interventions^58^, early identification of pre-diabetes among adolescents with obesity becomes increasingly important.

Although small, our study population was likely representative of Auckland’s ethnic and socioeconomic make-up, with relatively similar demographics when compared to national census data^59^. Both ethnicity and socioeconomic status are factors known to be associated with an increased risk of obesity and obesity-related diseases^60^. As we were able to recruit adolescents with obesity but not with any pre-diagnosed chronic conditions from the general population, our findings may be extrapolated to describe the health of adolescents with obesity in Auckland.

A strength of our study was our robust clinical assessments. In particular, accurate measurement of clinic BP is challenging, with wide variations due to many environmental factors^61^. 24hABPM, is a far more robust method to identify BP abnormalities compared to commonly used clinic devices^62^. Notably, pre-hypertension/hypertension was underdiagnosed when measured using the clinic BP monitor; only one in five was diagnosed to have elevated BP whereas with a 24hABPM, more than a third were reported to have elevated BP. Moreover, nearly a third of participants were diagnosed to have nocturnal prehypertension/hypertension which would have been undetected during daytime clinic BP measurements, and further emphasized the importance of undertaking BP monitoring over a 24-h period. Participants also underwent an OGTT which provided a more comprehensive assessment of glucose homeostasis and insulin sensitivity^53^.

In conclusion, we identified a high prevalence of undiagnosed comorbidities among adolescents with obesity. Of note, the high prevalence of metabolic syndrome in our study population emphasises the importance of screening adolescents with obesity for these metabolic complications. Obesity is a complex chronic condition that once established is not only difficult to treat, but requires life-long support^41^. As a result, it is undeniable that prevention of obesity should be the primary focus in this health crisis. However, for adolescents with established obesity, early identification of individuals with poor metabolic health and implementation of early targeted interventions are important, with the aim of preventing the development of overt cardiometabolic disease.

## Data Availability

The clinical data cannot be made available in a public repository according to the strict conditions of the study's ethics approval. Nonetheless, anonymized and de-identified data could be made available to other investigators upon bona fide request, and following all the necessary approvals (including ethics) of the detailed study proposal and statistical analyses plan. Any queries should be directed to Prof Wayne Cutfield (w.cutfield@auckland.ac.nz).
